# Prevalence and patterns of workplace violence against doctors of North India: a cross-sectional study

**DOI:** 10.3389/fpubh.2026.1653005

**Published:** 2026-05-04

**Authors:** Shaili Vyas, Abhinav Singh, Veena Boswal, Abhay Srivastava, Neha Sharma, Syed Esam Mahmood, Akash Krishali, Abhinav Bahuguna, Jayanti Semwal, Vipul Nautiyal

**Affiliations:** 1 Department of Community Medicine, Himalayan Institute of Medical Sciences, Swami Rama Himalayan University, Dehradun, Uttarakhand, India; 2 Department of Anaesthesiology, Graphic Era Institute of Medical Sciences, Dehradun, Uttarakhand, India; 3 Department of Family and Community Medicine, College of Medicine, King Khalid University, Abha, Saudi Arabia; 4 Department of Biostatistics, Himalayan Institute of Medical Sciences, Swami Rama Himalayan University, Dehradun, Uttarakhand, India; 5 Department of Radiation Oncology, CCRC, Himalayan Institute of Medical Sciences, Swami Rama Himalayan University, Dehradun, Uttarakhand, India

**Keywords:** healthcare security, hospitals, India, occupational health, physicians, Uttarakhand, verbal abuse, workplace violence

## Abstract

**Background:**

Workplace violence (WPV) against physicians represents an escalating global concern, with significant implications for India. This phenomenon substantially undermines the mental health, professional morale, and clinical performance of healthcare providers. A comprehensive understanding of the prevalence, patterns, and contextual factors associated with WPV is essential to develop evidence-based prevention and mitigation strategies.

**Methods:**

A cross-sectional study was conducted with 200 physicians (100 employed at a tertiary care teaching hospital and 100 in private healthcare settings) in Dehradun, Uttarakhand, North India. Data were collected using a pretested, self-administered questionnaire assessing experiences of workplace violence, contributing factors, physicians’ perceptions, and recommended preventive measures. Both descriptive and inferential statistical analyses were performed using SPSS version 23.

**Results:**

Workplace violence was reported by 75% of the respondents (95% confidence interval [CI]: 68.9–81.0%), with verbal abuse representing the most prevalent form (59.33%; 95% CI: 51.4–67.1%). Violence occurred more frequently among male physicians, particularly in private healthcare settings and surgical specialties. A majority of incidents occurred during junior residency and were perpetrated by patients’ relatives (68%) or organized groups (mobs). Notably, only 18% of cases were formally reported by police authorities.

**Conclusion:**

Workplace violence was highly prevalent among physicians in Uttarakhand, with verbal abuse and mob-related incidents constituting the predominant manifestations. Urgent interventions, including enhanced security measures, structured physician-patient communication programs, and stringent legal enforcement, are necessary to establish a safer and more supportive working environment for healthcare professionals.

## Introduction

The World Health Organization (WHO) defines violence as “the intentional use of physical force or power, threatened or actual, against oneself, another person, or against a group or community, which either results in or has a high likelihood of resulting in injury, death, psychological harm, maldevelopment, or deprivation” (1). Workplace violence (WPV) is specifically defined by the WHO as incidents in which staff are abused, threatened, or assaulted in circumstances related to their work, including travel to and from work sites, involving an explicit or implicit challenge to their safety, wellbeing, or health (2). Violence in occupational settings constitutes a critical public health hazard within healthcare facilities worldwide and represents an escalating concern in both developed and developing nations (3, 4).

Violence directed toward physicians is not geographically localized to any particular region or nation; rather, it represents a widespread phenomenon that occurs globally. Contemporary documentation through social media demonstrates the prevalence of such incidents, with substantive cases of violence against physicians emerging at a notable frequency and achieving rapid online dissemination (5).

Historically, individuals entering the medical profession pursued the noble objective of healing human suffering and were consequently regarded as benefactors of society. However, the progressive commercialization of medical practice has led to accusations against some practitioners pursuing financial gain and engaging in unethical conduct (6). This erosion of professional esteem has diminished traditional reverence according to physicians (7). While acknowledging that unethical practitioners do exist within the profession, the generalized perception that the entire medical community operates with questionable motives has fundamentally compromised the physician–patient relationship and contributed to escalating the victimization of healthcare providers (6). Inadequate healthcare service delivery coupled with increasing patient awareness regarding medical rights has generated heightened medical grievances and, in some cases, physical aggression toward healthcare providers (8). Contributing factors include insufficient medical workforce capacity and healthcare infrastructure for managing substantial patient volumes (9). Additionally, insufficiently developed and underfunded healthcare insurance systems restrict patient options and render healthcare economically inaccessible to the majority of individuals (9). Extended waiting periods, abbreviated as clinical consultation times, and deteriorating interpersonal relations between physicians and patients create tension when patient expectations remain unmet (7).

According to WHO estimates, between 8 and 38% of healthcare workers experience physical violence during their professional career (10). The Indian Medical Association (IMA) has reported that approximately 75% of physicians encounter physical or verbal violence during their lifetime (11). Given their roles as caregivers, physicians represent accessible targets for the expression of patients and attendant frustration. This phenomenon is attributable to multiple factors, including the commercialization of medical practice, insufficient government investment in healthcare, adverse media representation, elevated medical expenses, and diminished public confidence in medical professionals (8).

Violence in professional settings constitutes an urgent concern, given that affected physicians frequently develop serious psychological sequelae, including major depression, post-traumatic stress disorder, agoraphobia, and anxiety disorders (12). Addressing this occupational hazard requires a comprehensive investigation of the magnitude of the problem and its underlying causal mechanisms. A systematic analysis from the physician’s perspective would facilitate evidence-based recommendations for violence prevention strategies and workplace protection measures.

## Methodology

### Study design and setting

This cross-sectional study was conducted at the Himalayan Institute of Medical Sciences (HIMS), a tertiary care teaching hospital in Dehradun, Uttarakhand, North India. Permission from the institutional Ethics committee Himalayan Institute of Medical Sciences, Swami Rama Himalayan University Dehradun was obtained prior to the initiation of the study (via Ref No: SRHU/HIMS/RC/2025/206). The study population comprised both institutional and private healthcare practitioners.

Institutional participants included junior residents, senior residents, and consultant physicians who had been employed for a minimum of 6 months and had at least 6 months of clinical experience in departments with direct patient interaction, either in outpatient settings or inpatient wards. Private-sector participants included physicians holding MBBS, MD, or postgraduate diploma qualifications in clinical specialties, with a minimum of 6 months of independent practice experience in Dehradun.

### Sample size calculation

Owing to the absence of prior prevalence data from Uttarakhand regarding violence against physicians at the beginning of the study, we estimated the sample size based on the assumed perception of violence risk among physicians. We conservatively assumed that 50% of physicians were at an elevated risk of experiencing violence from patients or their attendants compared with other professions.

The sample size was calculated using the formula:


$$N=\frac{Z_{\alpha /2}^{2}\times P\times Q}{l^{2}},$$

where *P* represents the assumed proportion (50%), *Q* represents 1 – *P* (50%) with a 5% significance level (*α* = 0.05), and l represents the allowable error, defined as 15% of *P*. By substituting these values, we obtain:


$$N=\frac{{\left(1.96\right)}^{2}\times 50\times 50}{{\left(15\right)}^{2}}=171.$$

Accounting for an anticipated 10% non-response rate, 17 additional participants were recruited, yielding a target sample size of 188, which was rounded to 200 for operational convenience.

### Sampling strategy and participant selection

Two hundred physicians were recruited using simple random sampling from two distinct healthcare sectors: 100 from a tertiary care teaching hospital and 100 from the private healthcare sector. Private healthcare settings included private nursing facilities, independent private practices, group clinics, and corporate or private hospitals unaffiliated with medical colleges.

For tertiary care hospital participants, the sampling frame comprised a comprehensive roster of all junior residents, senior residents, and consultant physicians who met the inclusion criteria. Participants were selected using computer-generated random numbers. For private practice participants, the sampling frame was developed using official registries and local clinical documentation obtained from medical directories. Physicians meeting the inclusion criteria were randomly selected using computer-generated randomization.

The 200 participating physicians represented diverse clinical specialties to ensure comprehensive representation. The specialty distribution was as follows: surgical specialties [including Master of Surgery (MS) and surgical subspecialties], 45% of the sample; medical specialties [including Doctor of Medicine (MD) and nonsurgical branches], 36% of the sample; and postgraduate diploma holders and superspecialists (DM/MCh), 19% of the sample. This diverse specialization representation enhanced the external validity of the study and facilitated a comprehensive investigation of workplace violence patterns across clinical disciplines.

### Participant recruitment and informed consent

Written informed consent was obtained from all participants prior to data collection. The objectives and procedures of the study were explained to each participant. Participants were informed that study participation was entirely voluntary, that their identities and personal information would remain anonymous, and that confidentiality would be maintained rigorously throughout the study period. Participants were explicitly informed of their right to withdraw from the study at any time without consequences.

### Data collection instrument

A pretested self-administered questionnaire in English was distributed to all participants. The instrument was developed through a systematic literature review, expert consultation, focus group discussions, and pilot testing. The validation was established through face and content validity assessments. The questionnaire utilized Likert-scale responses to the appropriate items. A pilot study incorporating 10% of the participants from each group (20 participants per group) was conducted, and the questionnaire was subsequently refined based on the pilot results.

The questionnaire comprised six distinct sections:

Section A: Sociodemographic characteristics and professional profiles of physicians, including age, sex, qualifications, specialty, and years of experience.

Section B: Incidents of workplace violence and institutional administrative responses to such incidents.

Section C: Physician perceptions of factors associated with workplace violence, including patient-related, physician-related, and healthcare system factors.

Section D: Physician perspectives regarding personal safety and workplace security concerns.

Section E: Physician perceptions regarding patient attitudes and expectations toward the medical profession.

Section F: Physician recommendations and suggestions for preventing and mitigating workplace violence in healthcare settings.

### Measurement and classification of variables

Workplace violence severity was categorized into three distinct categories based on established classification systems (13):

1. Mild violence: verbal or emotional abuse, abusive gestures (including inappropriate staring, inadequate eye contact, mumbling, slurred or incoherent speech, facial expressions, or antagonistic eye contact), or verbal intimidation.
2. Moderate violence: intimidation, explicit threats, aggressive pacing, and threatening behavioral displays.
3. Severe violence: physical violence, sexual harassment, attacks with weapons, theft, or damage to personal or family property.

### Statistical analysis

Statistical analysis was performed using SPSS software (version 23, IBM, Chicago, United States). Descriptive statistics were calculated and are presented as frequencies and percentages. Associations between categorical variables were assessed using the chi-squared test. A multivariable logistic regression analysis was conducted to identify independent predictors of exposure to workplace violence. Graphs and tables were prepared for data visualization. Statistical significance was established at a *p*-value of <0.05, with 95% confidence intervals calculated for prevalence estimates.

## Results

### Demographic and professional characteristics

Table 1 presents the distribution of the participating physicians according to demographic and professional variables. The study sample comprised 55.5% male and 44.5% female physicians. The majority of participants (41.5%) were in the 31–40 year age group, followed by the 25–30 year age group (36.5%). A statistically significant association was found between age and sex (*p* < 0.05). Regarding educational qualifications, the majority of physicians (45%) held Master of Surgery (MS) degrees in surgical specialties, followed by those with medical specialties holding MD degrees (36%). Postgraduate diploma holders and superspecialists accounted for 19% of the sample.

**Table 1:** Sociodemographic characteristics of medical professionals (*N* = 200).

Age-group (in years)	Sex		Total (*N* = 200)
	Male *n* = 111(55.5%)*	Female *n* = 89(44.5%)*	
25–30	30(27.03)	43(48.32)	73(36.5)
31–40	54(48.65)	29(32.58)	83(41.5)
41–50	12(10.81)	12(13.48)	24(12)
>50	15(13.51)	5(5.62)	20(10)
Total	11(55.50)	89 (44.50)	200(100)
*χ*^2^ = 12.58, *p* = 0.005646^@^			
Professional degree			
MBBS	9(8.1)	5(5.7)	14(7)
Diploma	5(4.5)	7(7.9)	12(6)
MD	40(36)	32(35.9)	72(36)
MS	46(41.5)	44(49.4)	90(45)
DM/MCh	11(9.9)	1(1.1)	12(6)
Total	11(55.50)	89 (44.50)	200(100)
*χ*^2^ = 10.26, *p* = 0.04364^@^			

*Values are expressed as number (column percentage).

*χ*^2^ test applied; ^@^*p* < 0.05 considered statistically significant.

MBBS: Bachelor of Medicine and Bachelor of Surgery, MD: Doctor of Medicine, MS: Master of Surgery, DM: Doctorate of Medicine, MCh: Master of Chirurgiae.

### Clinical practice characteristics

Table 2 shows the distribution of physicians according to consultation duration and daily working hours. A majority of physicians (38%) spent 8–12 min per patient consultation. In private practice, 35.6% of male physicians most commonly allocated 12–15 minutes per patient, compared with 13.7% of female physicians. In contrast, physicians in teaching hospitals demonstrated more variable consultation patterns, with substantial proportions allocating either 8–12 min or more than 15 min per patient. This pattern indicates that private practice physicians generally allocate greater consultation time (12–15 min per patient) than teaching hospital colleagues.

**Table 2:** Working profile of medical professionals by type of healthcare facility (*N* = 200).

Working profile	Type of medical facility				Total (*N* = 200)
	Private health care facilities^†^ N1 = 100		Medical college hospital N2 = 100		
	Male n1 = 59(59)*	Female n2 = 41(41)*	Male n1 = 52(52)*	Female n2 = 48(48)*	
Average patient consultation time (minutes)					
2–5	6(10.16)	5(12.19)	7(13.46)	2(4.16)	20(10)
5–8	2(3.38)	1(2.43)	3(5.76)	2(4.16)	8(4)
8–12	22(25.42)	16(19.51)	18(17.30)	20(18.75)	76(38)
12–15	21(35.59)	13(13.70)	14(26.92)	4(8.33)	52(26)
>15	8(13.55)	6(14.63)	10(19.23)	20(41.66)	44(22)
*χ*^2^ = 12.77, *p* = 0.025^@^					
Working hours per day (hours)					
4–8 h	12(20.33)	16(39.02)	19(36.53)	11(22.91)	58(29)
9–12 h	37(62.71)	18(43.90)	24(46.15)	17(35.41)	96(48)
13–16 h	6(10.16)	2(4.87)	5(9.61)	11(22.91)	24(12)
17–20 h	0(0)	0(0)	2(3.84)	1(2.08)	3(1.5)
21–24 h	4(6.77)	5(12.19)	2(3.84)	8(16.66)	19(9.5)
*χ*^2^ = 5.5, *p* = 0.1384					
Number of patients attended/day					
20–40	18(30.5)	6(14.7)	10(19.2)	6(12.4)	40(20)
40–60	20(33.9)	21(51.2)	15(28.8)	8(16.7)	64(32)
>60	21(35.6)	14(34.1)	27(52)	34(70.9)	96(48)
*χ*^2^ = 8.9, *p* = 0.0348^@^					

*Values are expressed as number (column percentage).

*χ*^2^ test applied for comparison; ^@^*p* < 0.05 considered statistically significant.

^†^Private healthcare facilities include private nursing homes, standalone private clinics, polyclinics, and non-teaching private hospitals.

**Figure 1:**
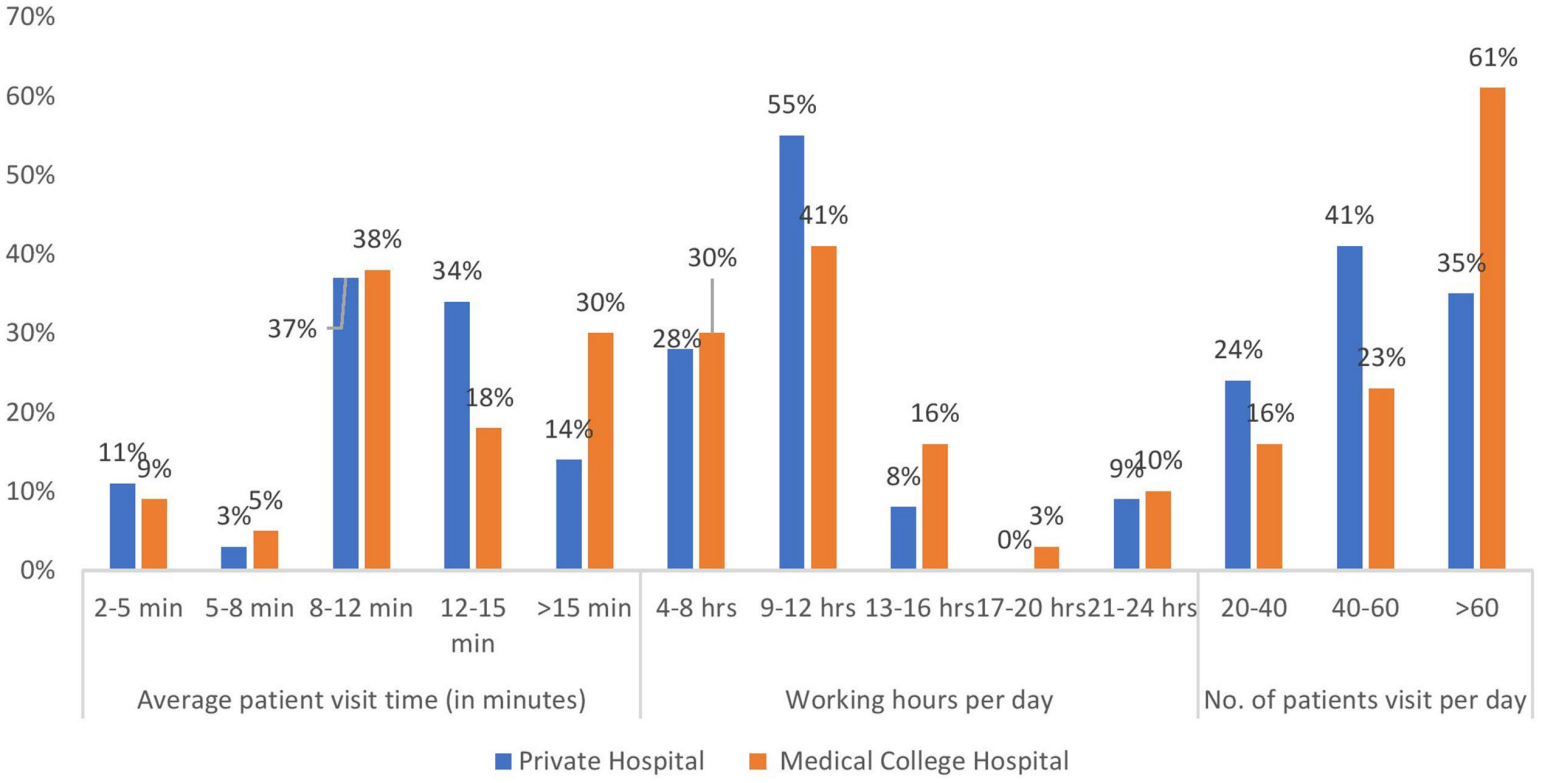
Distribution of study subject by working profile.

Approximately half of all physicians (48%) worked 9–12 h daily, whereas approximately 10% (9.5%) worked extended shifts of 21–24 h daily. The number of patients seen daily varied significantly across the samples (*χ*^2^ = 8.9, *p* = 0.0348). Nearly half of the respondents (48%) reported managing more than 60 patients daily, with a particularly high rate among female physicians in teaching hospitals (70.9%). Approximately one-third of the physicians (32%) managed 40–60 patients daily, while 20% managed 20–40 patients daily (Figure 1).

### Prevalence and characteristics of workplace violence

**Figure 2:**
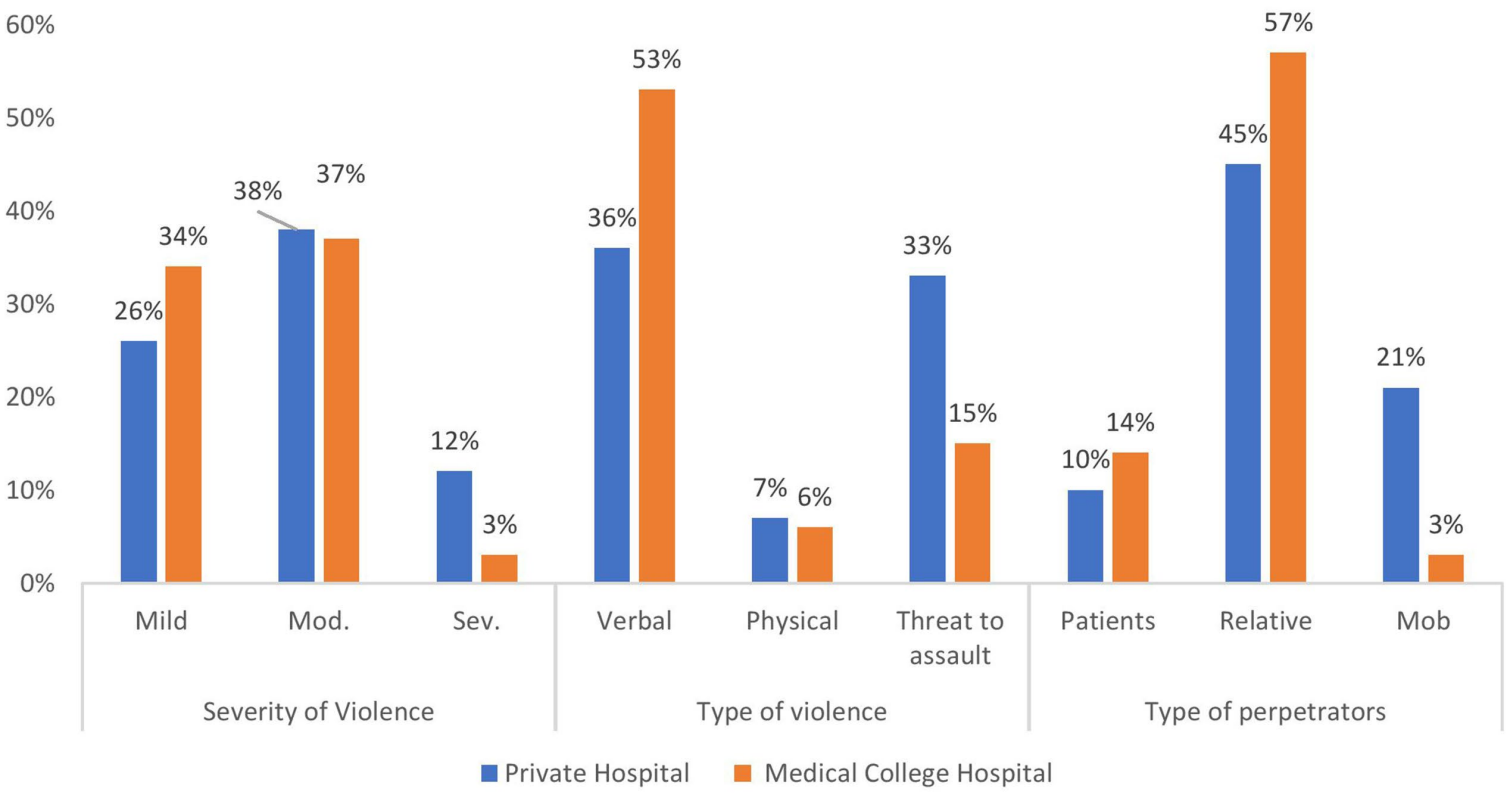
Distribution of study subject by workplace violence.

Figure 2 depicts the distribution of workplace violence among healthcare professionals in private hospitals and medical college hospitals. Mild and moderate violence were more commonly reported in both settings, while severe violence was relatively higher in private hospitals (12%) compared to medical college hospitals (3%). Verbal violence was the predominant type, especially in medical college hospitals (53%), and relatives were the most frequent perpetrators in both settings, followed by mobs and patients Overall, 75% of the respondents (95% CI, 68.9–81.0%) reported experiencing workplace violence during their professional careers. Table 3 illustrates the distribution of workplace violence experiences according to the type of healthcare setting. Male physicians in private healthcare settings reported the highest prevalence of exposure to violence (31.33%) compared with their counterparts in teaching hospitals. Approximately half of the respondents reported experiencing a moderate degree of violence. Severe violence was reported by 23.40% of male physicians in private healthcare settings, and the association between violence severity and type of healthcare facility was statistically significant (*p* < 0.001) (Table 3).

**Table 3:** Prevalence and characteristics of workplace violence among doctors (*n* = 150).

Severity of workplace violence^$^	Type of medical facility				Total (*N* = 150) % (95%CI)*
	Private health care facilities N1 = 76		Medical college hospital N2 = 74		
	Male n1 = 47(61.84)	Female n2 = 29(38.16)	Male n1 = 35(47.30)	Female n2 = 39(52.70)	
Mild	14(29.78)	12(41.73)	11(31.42)	23(58.97)	60 (40) (32.2–47.8%)
Moderate	22(46.80)	16(55.71)	21(60)	16(41.03)	75 (50) (42.0–58.0%)
Severe	11(23.40)	1(3.44)	3(8.58)	0(0)	15 (10) (5.2–14.8%)
*χ*^2^ = 6.454, *p* = 0.0396^@^					
Type of violence experienced					
Verbal	18(38.29)	18(62.06)	18(51.42)	35(89.74)	89 (59.33)(51.4–67.1%)
Physical	6(12.76)	1(3.44)	5(14.28)	1(2.56)	13 (8.66) (27.1–42.2%)
Threat to assault	23(48.93)	10(34.48)	12(34.28)	3(7.69)	52 (34.66) (4.2–13.2%)
*χ*^2^ = 10.05, *p* = 0.00657^@^					
Type of perpetrators involved					
Patients	8(17.02)	2(6.89)	6(17.14)	8(20.51)	24 (16) (60.5–75.5%)
Relative	21(44.68)	24(82.75)	28(80)	29(74.35)	102 (68)(10.1–21.9%)
Mob	18(38.29)	3(10.34)	1(2.85)	2(5.12)	24 (16) (10.1–21.9%)
*χ*^2^ = 15.55, *p* = 0.000419^#^					

*Prevalence estimates are presented with 95% confidence intervals (CI).

*χ*^2^ test applied; ^@^*p* < 0.05 significant, #*p* < 0.001 highly significant.

^$^Severity of violence has been categorized into three types (13):

(a) Mild violence-verbal/emotional abuse, abuse by gestures (staring/lack of eye contact/mumbling/slurred or incoherent speech/ making faces/showing eyes, giving bad looks).

(b) Moderate violence-intimidation/Threat/Pacing.

(c) Severe violence-Physical violence/sexual harassment/weaponry attacks/Theft/Damage to family or property.

Verbal violence was the most prevalent form of violence, affecting 59.33% of respondents, with statistically significant variation across subgroups (*p* < 0.05) (Table 3). In the majority of incidents (68%), the perpetrator was a patient’s relative, whereas 38.29% of male physicians in private healthcare settings reported assault by organized groups (mobs) (Table 3). Severe violence was experienced by 23.30% of the male physicians in private healthcare settings (Table 3).

### Violence patterns according to clinical specialty

**Figure 3:**
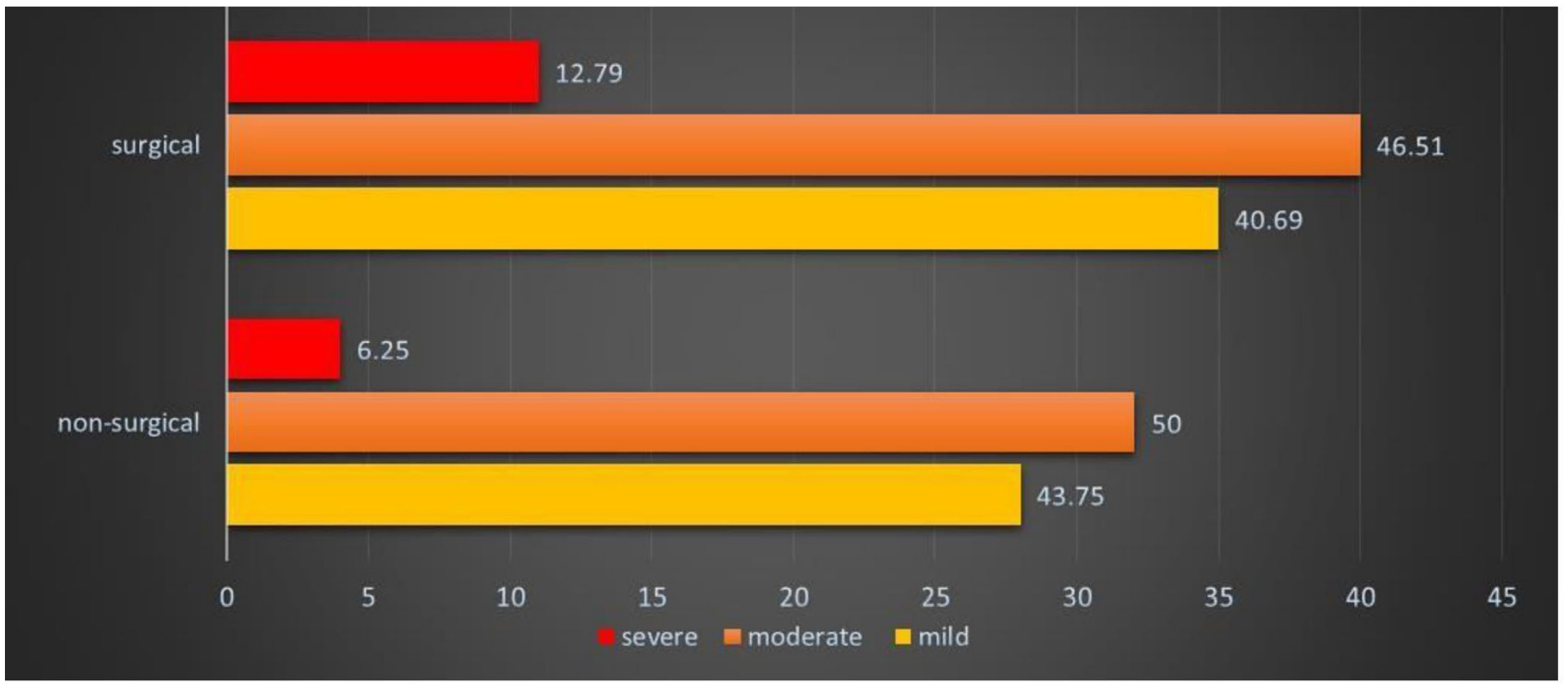
Distribution of workplace violence severity across medical specialties.

Figure 3 illustrates the distribution of violence according to clinical specialty and severity. Mild violence (43.75%) and moderate violence (50%) were predominant in non-surgical clinical specialties, whereas severe violence (12.79%) was predominant in surgical specialties. These data suggest differential vulnerability according to the clinical discipline.

### Timing of violence exposure during professional career

**Figure 4:**
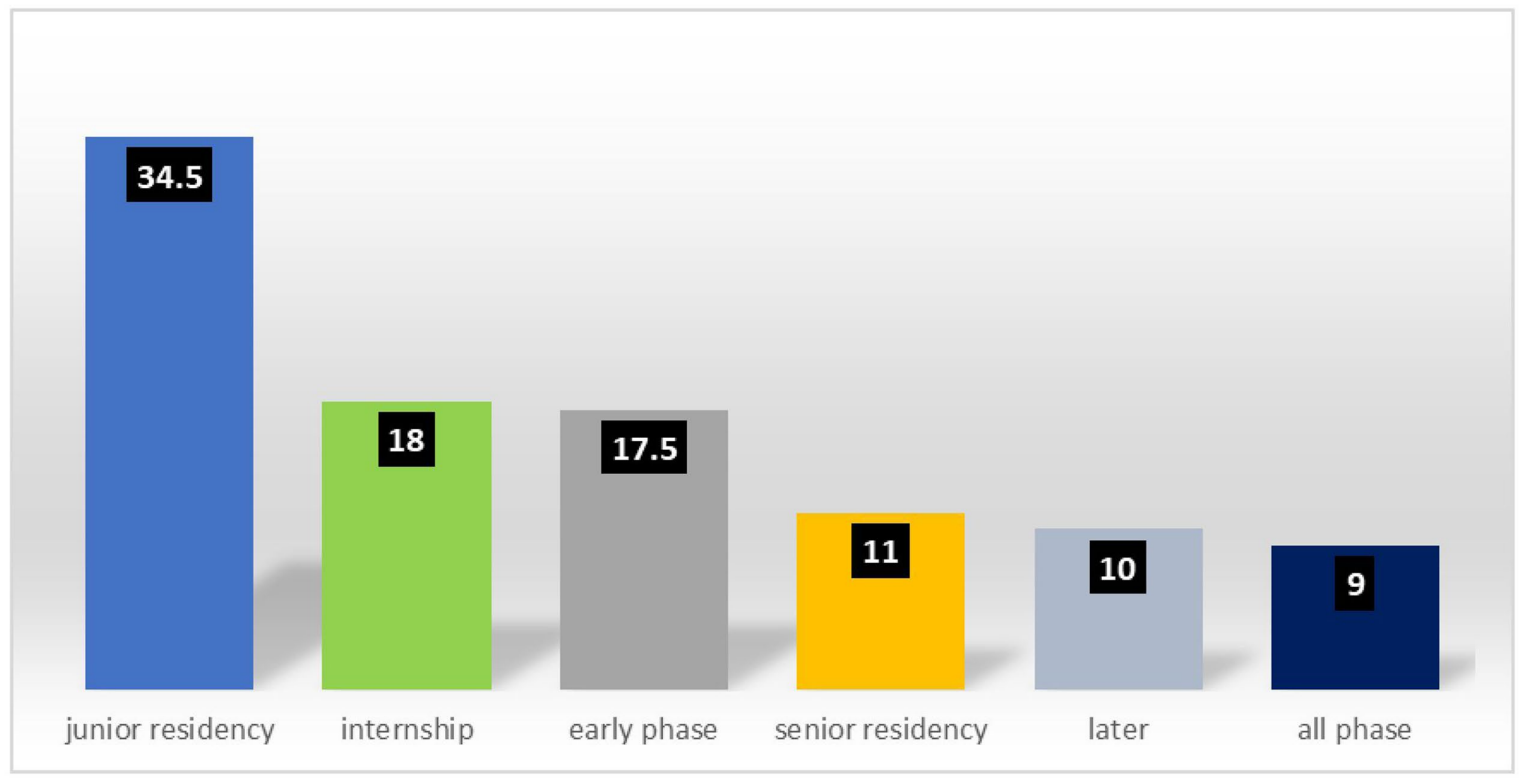
Phase of medical career during which doctors experienced workplace violence.

Figure 4 shows the temporal distribution of workplace violence experiences during professional careers. The highest prevalence of exposure to violence occurred during junior residency (34.5%), followed by the internship period (18%). These findings indicate a particular vulnerability during early career stages.

### Reporting of workplace violence incidents

**Figure 5:**
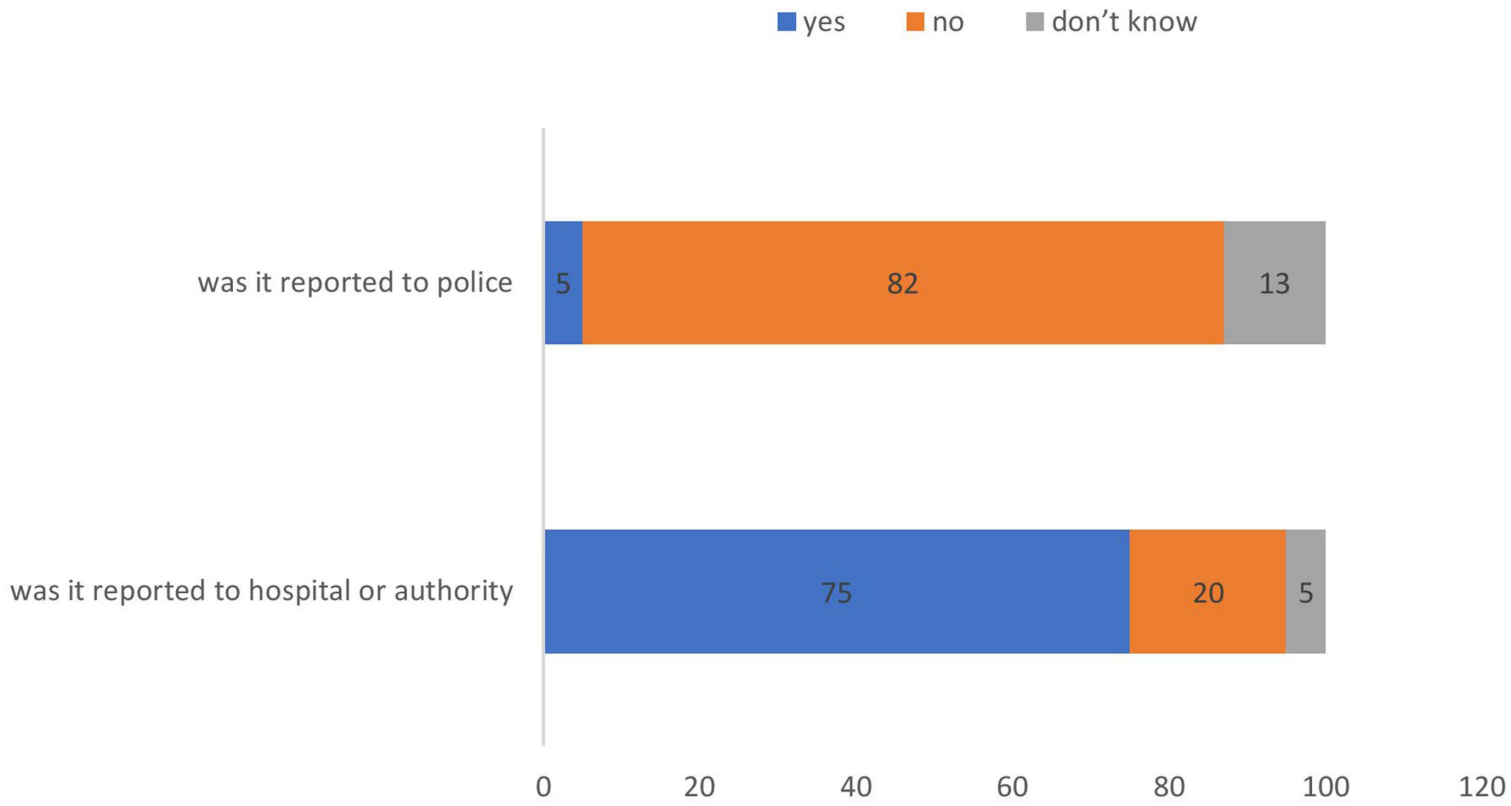
Actions taken following workplace violence and reporting to police.

Figure 5 shows the reporting patterns of workplace violence incidents. Although 75% of the incidents were formally reported to hospital authorities, the majority (82%) were not reported to police authorities. This substantial reporting gap in law enforcement suggests underreporting and inadequate utilization of formal legal channels.

### Multivariable analysis of predictors

A multivariate logistic regression analysis identified several statistically significant predictors of exposure to workplace violence. The average consultation time per patient was a significant positive predictor (OR = 1.09, *p* = 0.012), indicating that increased consultation duration was associated with higher odds of exposure to violence. In contrast, physicians with on-call duty responsibilities demonstrated significantly lower odds of violence exposure than those without such duties (OR = 0.30, *p* = 0.030).

Among educational qualifications, diploma holders demonstrated higher odds of violence exposure than the reference category (OR = 2.36, *p* = 0.050), although overall educational qualifications did not achieve statistical significance as a group variable. Notably, the timing of initial exposure to violence during junior residency was a significant independent predictor (*p* = 0.003) (Figure 4). Compared with the reference category, physicians experiencing their first workplace violence episode during junior residency demonstrated substantially elevated odds of violence exposure, with an 11.67-fold increase (Exp[B] = 11.667, *p* = 0.030).

In contrast, age, sex, healthcare facility type, and daily working hours were not identified as significant independent predictors of workplace violence in the multivariable model.

## Discussion

### Study population characteristics

Participants in this investigation comprised two distinct physician groups: (1) physicians employed by teaching hospital institutions (male and female) and (2) physicians engaged in private healthcare practice (male and female). The majority of participating physicians fell within the 31–40-year age group. The sex distribution was 55.5% male and 44.5% female physicians, which contrasts with data from a study in Aurangabad that reported 87.8% male and 12.2% female participation (12).

### Working hours, patient load, and consultation duration

In the present study, the majority of physicians (48%) maintained working schedules of 9–12 h daily, with 26% reporting average consultation times of 12–15 min per patient. In contrast, a study in China reported substantially shorter consultation durations, with an average visit duration of 5–9 min, affecting 41.1% of physicians (14). The present study demonstrated that 48.5% of physicians managed more than 60 patients daily, reflecting a substantial clinical workload. This high patient volume combined with extended working hours suggests considerable occupational stress and potential compromise in care quality and physician wellness.

The elevated patient-to-physician ratio observed in this study likely reflects several factors, including substantial demand for medical services, physician workforce shortages, and inadequate healthcare infrastructure and resources. These systemic deficiencies may necessitate workforce expansion, enhanced productivity mechanisms, or patient load redistribution to optimize clinical outcomes and physician wellbeing.

### Prevalence of workplace violence

Of the 200 participants, 150 (75%) reported experiencing at least one workplace violence incident within their professional career, establishing a prevalence rate of 75%. This finding is consistent with those of comparable studies conducted in South Asia. Imran et al. (15) in Lahore reported a prevalence of 74%, Singh et al. (16) in Agra reported 69.5%, and Pund et al. (12) in Aurangabad reported 63%, demonstrating regional consistency in estimates of violence prevalence.

Approximately half (50%) of the respondents in the present study reported experiencing moderate violence. This contrasts with the findings of Ahmed et al. in Pakistan, who reported that the majority of physicians experienced mild assault (85.11%), followed by moderate (62%), and severe (38.1%) incidents (13).

### Types of violence experienced

Verbal aggression was reported by 59.33% of physicians during the preceding 12 months, whereas threats of assault were experienced by 34.66% of the respondents. The predominance of verbal violence observed in this investigation is consistent with the findings from multiple comparable studies. Studies conducted in Agra by Singh et al. (16), in Haryana by Vaishali et al. (17), in Gujarat by Sharma et al. (18), in Delhi by Gohil et al. (19) and Aurangabad in Pund et al. (12) reported verbal violence as the most prevalent manifestation, with prevalence rates varying from 55.3 to 85% across different investigations.

Female physicians in both private settings (62.06%) and teaching hospitals (89.74%) reported higher prevalence of verbal abuse than their male counterparts. In contrast, threats and physical violence were more prevalent among male physicians. Notably, mob attacks were substantially more common among male physicians in private healthcare settings (38.29%) than female physicians (10.34%). A marginally higher prevalence of verbal violence among male physicians was reported by Vaishali et al. (17) in Haryana.

The incidence of physical violence in the present study (8.66%) was substantially lower than that reported in other comparable studies. Vaishali et al. (17) in Haryana reported physical violence in 20.9% of participants, whereas Singh et al. in Agra reported it in 47.2% (16). These regional variations may reflect cultural and social differences in conflict resolution patterns. The Uttarakhand population, historically known as “Pahssaris” (mountain dwellers), traditionally demonstrates peaceful and nonviolent behavioral patterns. Additionally, cultural norms in Indian society accord with women’s distinctive social status, and violent aggression toward women is generally culturally less acceptable than aggression toward men.

### Perpetrators of workplace violence

According to the study data, patients’ relatives perpetrated 66.66% of violent incidents, whereas organized groups (mobs) accounted for 19.33% of incidents. Comparable findings were reported by Gohil et al. (19) in Delhi, who identified patients’ attendants as primary perpetrators in 80% of cases, and by Singh et al. (16) in Agra, who reported that 69.3% of violent incidents were perpetrated by patients’ attendants. In contrast, Ahmed et al. reported that 63% of violent incidents in Pakistan involved single perpetrators acting independently rather than in organized groups (13).

Research by de-San-Segundo et al. (20) suggests differential perpetrator profiles according to healthcare settings, with patients representing primary perpetrators in primary care environments, whereas attendants constitute primary perpetrators in hospital settings. Wu et al. (14), in China, noted that violence frequently emerges from patient escorts’ perceptions—whether accurate or inaccurate—of healthcare provider shortcomings or errors. In certain circumstances, dissatisfied patients and family members engage criminal organizations prepared to undertake extreme actions to pressure hospitals to provide compensation (14).

### Temporal patterns: violence during career stages

The present investigation demonstrated that workplace violence experiences peaked during junior residency (34.5%), followed by internship (18%). These findings align with the research by Pund et al. (12), who similarly identified increased violence occurrence during the initial career stages. Sen and Honavar (21) emphasized that hospital operational structures depend heavily on resident physicians, who consequently experience an elevated vulnerability to violence.

Junior residents and interns frequently serve as initial patient contact points, directly interacting with the patients and their families. However, early career physicians often lack the requisite experience to adequately address complex patient inquiries. Furthermore, patients and families frequently fail to accord residents and interns with the professional respect and recognition afforded to more experienced physicians, thereby engendering conflict. The combination of limited experience and reduced emotional maturity in junior physicians may contribute to the ineffective management of perceived slights and heightened susceptibility to violence.

### Reporting of violence incidents and administrative response

Although 75% of the respondents reported workplace violence incidents to hospital authorities, 82% indicated that such incidents were not reported to the police. This pattern is consistent with findings from Ahmed et al. in Pakistan, who reported that, while approximately 74% of physicians discussed incidents with others, 88% received no organizational support from the hospital administration (13). In Delhi, Anand et al. (22) found that only 44.2% of incidents were reported to hospital authorities, whereas Singh et al. (16) reported that only 9.4% of cases were forwarded to police authorities. Kumar et al. (23) noted that no formal police investigations were initiated in their study population.

Collectively, these data indicate that healthcare institutions have systematically failed to ensure physician safety and have inadequately utilized legal mechanisms for perpetrator accountability. A publication in The Lancet suggests that 70–80% of cases of violence against healthcare workers remain officially unreported (6). Nagpal (5) documented that, although one-third of the physicians had experienced verbal or physical assault in the preceding year, more than half (52%) of the physicians chose not to formally report the incident.

### Workplace violence according to clinical specialty

Surgical specialties demonstrated greater vulnerability to workplace violence, with moderate violence reported by 46.51% and severe violence by 12.79% of respondents compared with non-surgical specialties, in which 50% reported moderate violence and 6.25% reported severe violence. Kumar et al. (23) reported that physicians in radiodiagnostic specialties encountered fewer incidents of violence than those in other specialties. In contrast, Stuart (24) found that mental health physicians experienced the highest violence risk, whereas Morrison et al. (25) documented substantial violence risk among psychiatrists and emergency medicine physicians.

The elevated risk of violence in surgical specialties may reflect the nature of surgical practice, wherein critical, time-sensitive decisions regarding patient outcomes necessitate rapid intervention and immediate engagement with patient family members immediately following procedures. In contrast, medical specialties typically involve prolonged treatment courses, wherein therapeutic effects manifest gradually over extended periods, potentially allowing greater opportunities for therapeutic alliance development and conflict resolution.

### Limitations

The decision to estimate the sample size based on perceived prevalence rather than established regional data reflected the absence of published prevalence estimates from Uttarakhand at study initiation. However, this approach introduced a potential recall bias, as participant responses may have been influenced by memory limitations or subjective interpretations of historical experiences. Future investigations should utilize baseline prevalence estimates derived from preliminary surveys or contemporary regional studies to enhance the precision of sample size calculation and to minimize potential bias when such data become available.

## Conclusion

This investigation demonstrated that workplace violence against physicians is highly prevalent in North India, particularly manifesting as verbal abuse, with notable gender- and specialty-related patterns. Female and junior physicians experienced increased verbal aggression, whereas male physicians in surgical specialties and private healthcare settings experienced more severe mob-related violence incidents. A majority of violent incidents were perpetrated by patients’ relatives, and a substantial proportion of cases remained unreported to law enforcement authorities. Key contributing factors include overburdened healthcare systems, compromised physician–patient communication, and inadequate public confidence in the medical profession.

Comprehensive preventive strategies should include multifaceted interventions incorporating enhanced hospital security infrastructure, structured communication and conflict resolution training programs, systematic public awareness and health literacy initiatives, and the implementation of stringent legal protection and accountability mechanisms. Institutional commitment to violence prevention, coupled with government support for healthcare worker protection, is essential for establishing and maintaining a safer, more supportive, and therapeutically beneficial work environment for physicians and other healthcare professionals.

## Data Availability

The raw data supporting the conclusions of this article will be made available by the authors, without undue reservation.
